# Supplementary figures and images for: Stochastic bond dynamics facilitates alignment of malaria parasite at erythrocyte membrane upon invasion
Source: eLife. 2020 May 18;9:e56500. doi: 10.7554/eLife.56500 (PMC7269671; doi:10.7554/eLife.56500)

# Experiments

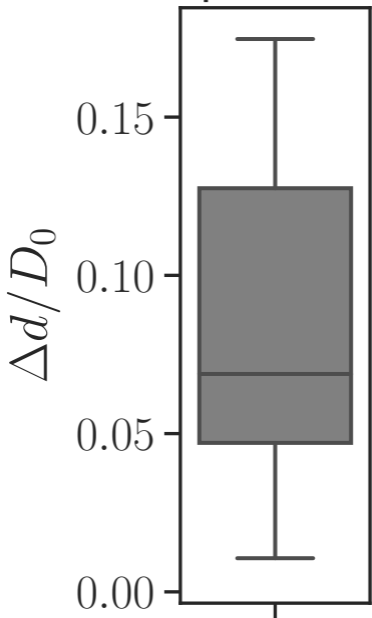

Supplement: Figure 2—source data 1. [file elife-56500-fig2-data1.zip › Fig2_b_experiments.pdf]

# Simulations

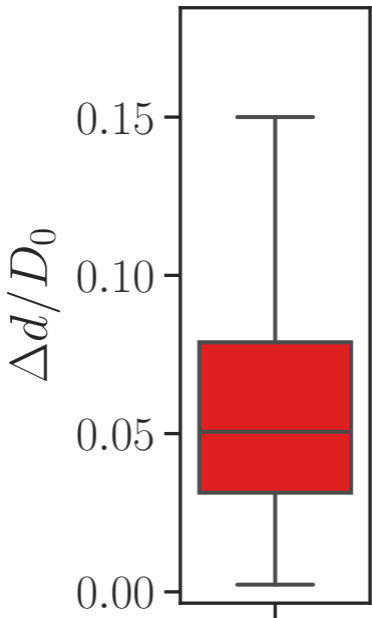

Supplement: Figure 2—source data 1. [file elife-56500-fig2-data1.zip › Fig2_b_simulations.pdf]

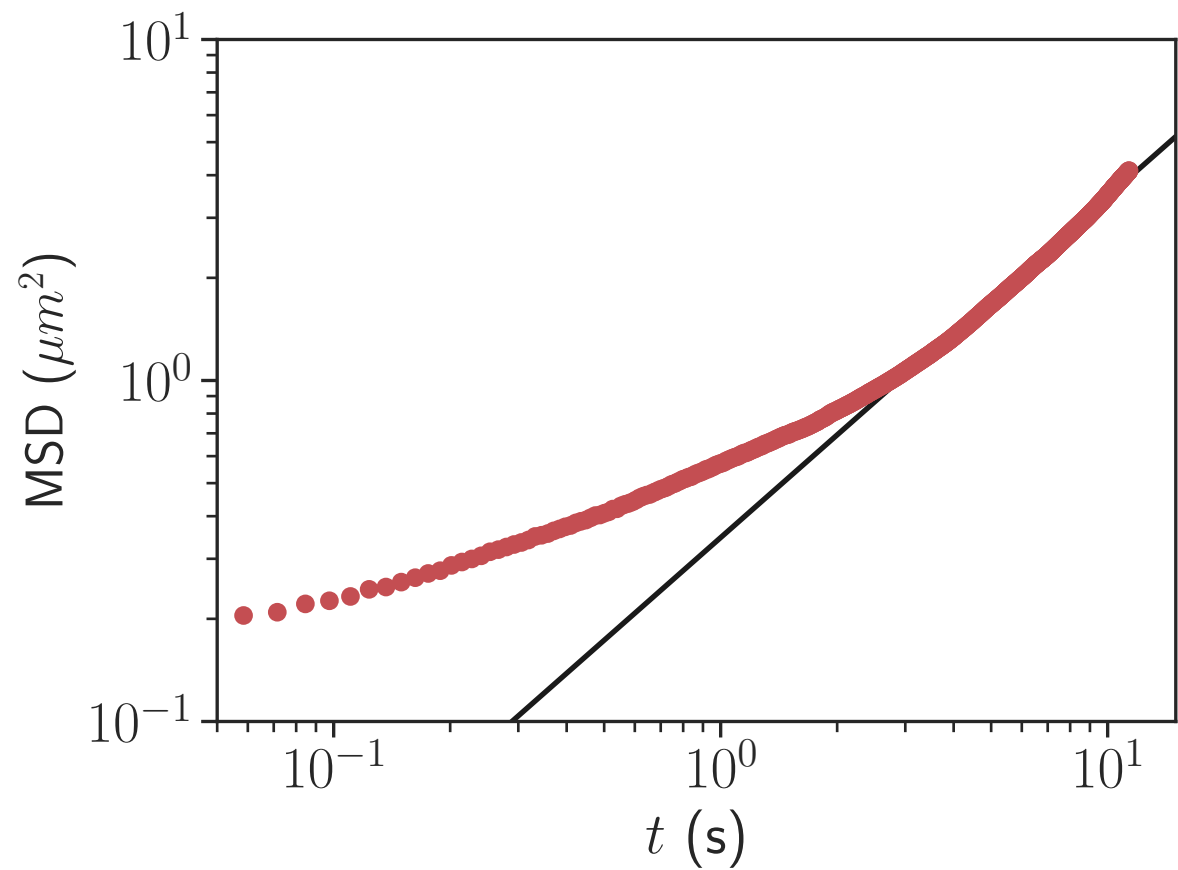

Supplement: Figure 2—source data 1. [file elife-56500-fig2-data1.zip › Fig2_c.pdf]

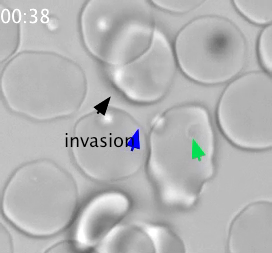

Supplement: Figure 2—source data 1. [file elife-56500-fig2-data1.zip › pictures/output_0051.png]

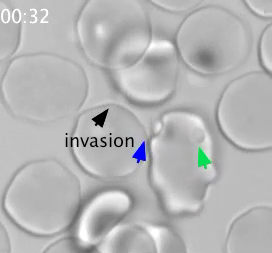

Supplement: Figure 2—source data 1. [file elife-56500-fig2-data1.zip › pictures/output_0045.png]

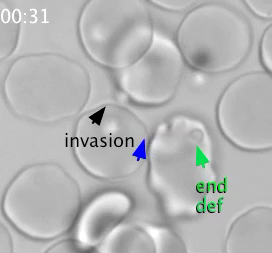

Supplement: Figure 2—source data 1. [file elife-56500-fig2-data1.zip › pictures/output_0044.png]

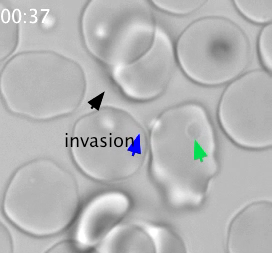

Supplement: Figure 2—source data 1. [file elife-56500-fig2-data1.zip › pictures/output_0050.png]

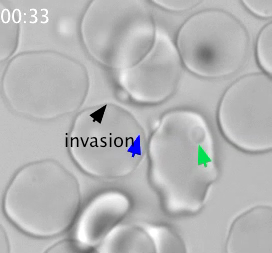

Supplement: Figure 2—source data 1. [file elife-56500-fig2-data1.zip › pictures/output_0046.png]

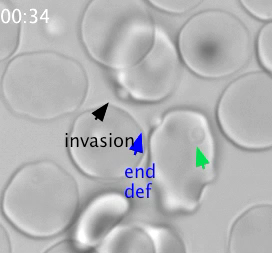

Supplement: Figure 2—source data 1. [file elife-56500-fig2-data1.zip › pictures/output_0047.png]

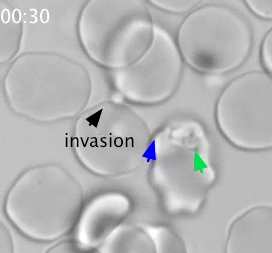

Supplement: Figure 2—source data 1. [file elife-56500-fig2-data1.zip › pictures/output_0043.png]

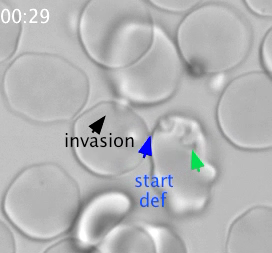

Supplement: Figure 2—source data 1. [file elife-56500-fig2-data1.zip › pictures/output_0042.png]

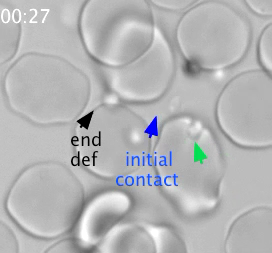

Supplement: Figure 2—source data 1. [file elife-56500-fig2-data1.zip › pictures/output_0040.png]

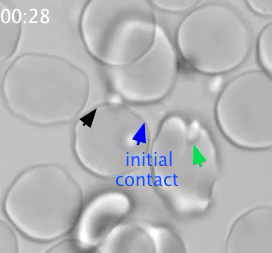

Supplement: Figure 2—source data 1. [file elife-56500-fig2-data1.zip › pictures/output_0041.png]

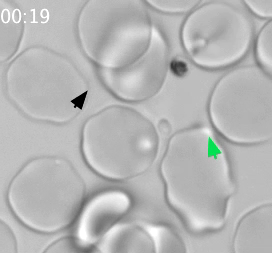

Supplement: Figure 2—source data 1. [file elife-56500-fig2-data1.zip › pictures/output_0032.png]

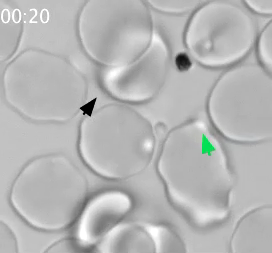

Supplement: Figure 2—source data 1. [file elife-56500-fig2-data1.zip › pictures/output_0033.png]

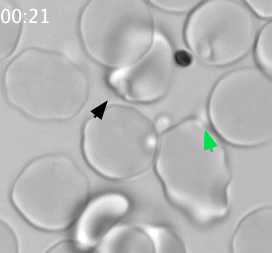

Supplement: Figure 2—source data 1. [file elife-56500-fig2-data1.zip › pictures/output_0034.png]

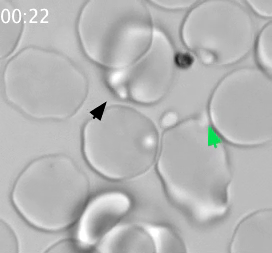

Supplement: Figure 2—source data 1. [file elife-56500-fig2-data1.zip › pictures/output_0035.png]

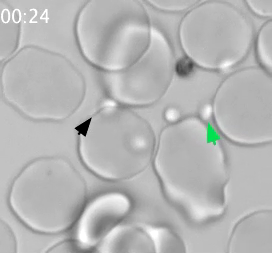

Supplement: Figure 2—source data 1. [file elife-56500-fig2-data1.zip › pictures/output_0037.png]

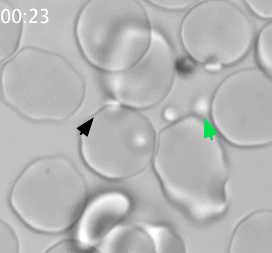

Supplement: Figure 2—source data 1. [file elife-56500-fig2-data1.zip › pictures/output_0036.png]

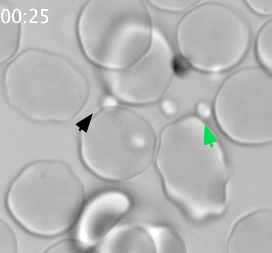

Supplement: Figure 2—source data 1. [file elife-56500-fig2-data1.zip › pictures/output_0038.png]

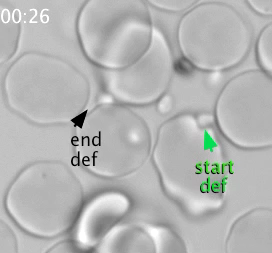

Supplement: Figure 2—source data 1. [file elife-56500-fig2-data1.zip › pictures/output_0039.png]

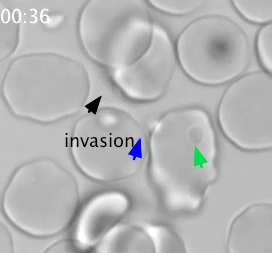

Supplement: Figure 2—source data 1. [file elife-56500-fig2-data1.zip › pictures/output_0049.png]

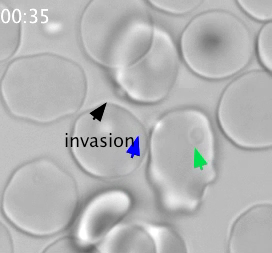

Supplement: Figure 2—source data 1. [file elife-56500-fig2-data1.zip › pictures/output_0048.png]
